# Drastic Dependence of the pH Sensitivity of Fe_2_O_3_-Bi_2_O_3_-B_2_O_3_ Hydrophobic Glasses with Composition

**DOI:** 10.3390/ma8125480

**Published:** 2015-12-10

**Authors:** Tadanori Hashimoto, Fumiya Murayama, Masashi Nakao, Hiroyuki Nasu, Atsushi Ishihara, Yuji Nishio

**Affiliations:** 1 Division of Chemistry for Materials, Graduate School of Engineering, Mie University, 1577 Kurimamachiya-Cho, Tsu City, Mie Prefecture 514-8507, Japan; mieuniv@me.com (F.M.); 415M338@m.mie-u.ac.jp (M.N.); nasu@chem.mie-u.ac.jp (H.N.); ishihara@chem.mie-u.ac.jp (A.I.); 2 HORIBA, Ltd., 2 Miyanohigasi, Kisshoin, Minami-Ku, Kyoto 601-8510, Japan; yuji.nishio@horiba.com

**Keywords:** Fe_2_O_3_-Bi_2_O_3_-B_2_O_3_ glasses, pH electrodes, hydrophobicity

## Abstract

Fe_2_O_3_-Bi_2_O_3_-B_2_O_3_ (FeBiB) glasses were developed as novel pH responsive hydrophobic glasses. The influence of the glass composition on the pH sensitivity of FeBiB glasses was investigated. The pH sensitivity drastically decreased with decreasing B_2_O_3_ content. A moderate amount of Fe_2_O_3_ and a small amount of B_2_O_3_ respectively produces bulk electronic conduction and a pH response on glass surfaces. Because the remaining components of the glass can be selected freely, this discovery could prove very useful in developing novel pH glass electrodes that are self-cleaning and resist fouling.

## 1. Introduction

Important customer issues in pH measurement are a decrease in pH sensitivity and an increase in pH response time, which mainly arise from the fouling due to contamination of the responsive glass membrane and liquid junction, and from the change in concentration of the internal liquid [1]. To avoid these issues, customers have to maintain their pH glass electrodes. This is troublesome, especially in industrial uses, because it is not easy to remove the accumulated stain from pH glass electrodes. For this reason, we have developed novel pH glass electrodes, such as TiO_2_-P_2_O_5_ (TP) glasses [2,3,4,5], with a self-cleaning property based on photocatalytic activity and photo-induced hydrophilicity [6]. TP glasses with low electrical resistivity gave a high pH sensitivity and short pH response time.

On the other hand, we have also reported that Bi_2_O_3_-B_2_O_3_ (BiB) glasses show hydrophobicity (contact angle of 90°) so far [7]. Materials with hydrophobicity have been used for anti-fouling and anti-fogging [8]. Glasses with hydrophobicity such as BiB glasses may be candidates of pH glass electrodes with an anti-fouling property based on their hydrophobicity, because pH measurement is basically carried out for aqueous solutions. The anti-fouling effect based on hydrophobicity may become remarkable, when pH of products and waste is monitored in fluid system as in industrial uses. Electric resistivity lower than 10^10^ Ω·cm, which is a representative value for commercially available pH glass electrode, is desirable in practical use. However, BiB glasses are undesirable for pH glass electrodes because of the relatively high electric resistivity (>10^11^ Ω·cm) [9]. It is well known that addition of transition metal oxides into glass composition causes the electronic conduction to the glasses as in TiO_2_-P_2_O_5_ glasses [6]. V_2_O_5_ and Fe_2_O_3_ are the most familiar additives as an origin of electronic conduction. Accordingly, Fe_2_O_3_-Bi_2_O_3_-B_2_O_3_ (FeBiB) glasses have been developed as novel pH responsive glasses with an anti-fouling property based on their hydrophobicity [10]. The electrical resistivity and pH response time of FeBiB glasses decreased with increasing Fe_2_O_3_ content, while their pH repeatability for standard solutions increased with increasing Bi_2_O_3_ content. FeBiB glasses showed a pH sensitivity close to that of commercial pH responsive glass, and shorter pH response time than that of a commercial glass. The contact angle for water of FeBiB glasses was relatively high (about 90°), similar to BiB glasses, and tended to increase slightly with increasing Bi_2_O_3_ content, regardless of Fe_2_O_3_ content. In this sense, we consider that Fe_2_O_3_ and Bi_2_O_3_ play an important role in electrical conduction in the bulk glass, and in the pH response and hydrophobicity, respectively. Such novel lithium-free nonsilicate pH responsive glasses are also expected to show a short pH response time, because they are a new type of pH glass electrodes based on “electronic conduction” that is different from the “ionic conduction” present in commercial lithium silicate glasses.

In the present study, the influence of Bi_2_O_3_ (or B_2_O_3_) content on the pH responsivity, electrical resistivity, and hydrophobicity (contact angle to water) for 20Fe_2_O_3_·*y*Bi_2_O_3_·(80 − *y*)B_2_O_3_ (20Fe*y*BiB, *y* = 70–80 mol %) glasses was investigated in order to reveal the role of each glass component.

## 2. Experimental

20Fe_2_O_3_·*y*Bi_2_O_3_·(80 − *y*)B_2_O_3_ (20Fe*y*BiB, *y* = 79, 79.5, 79.7, 79.8, 79.9 and 80 mol %) glasses were produced by a conventional melt-quenching method in 30 g batches under the following preparation conditions: melting at 1100 °C for 1 h, annealing at 350 °C for 1 h. Fe_2_O_3_ (99.9%, Kojundo Chemical Lab. Co., Ltd., Sakado, Japan), Bi_2_O_3_ (99.9%, Kojundo Chemical Lab. Co., Ltd., Sakado, Japan) and B_2_O_3_ (90%, guaranteed reagent grade, Nacalai Tesque, Inc., Kyoto, Japan) were used as raw materials. For example, 20Fe_2_O_3_·70Bi_2_O_3_·10B_2_O_3_ glass is abbreviated to 20Fe70BiB as a sample name in Table 1.

Potentiometric measurements for the 20Fe*y*BiB glasses was carried out at 25 °C, at time intervals of 3 s and 0.5 s using a pH meter F-73 (HORIBA, Ltd., Kyoto, Japan) and a portable multi logger ZR-RX20 (OMRON Corp., Kyoto, Japan) equipped with a handmade cell with a glass membrane of 1mm thickness, respectively. The details of the characterization of the pH responsivity (pH sensitivity and pH response time) were described in [8].

The direct current (DC) electrical resistivity of 20Fe*y*BiB glasses with ~1 mm thickness and an Ag electrode of 6 mm φ on both sides was measured at 25 °C using a super megohm meter SM-8215 (HIOKI E.E. Corp., Ueda, Japan). The contact angle for ~2 μL of water on 20Fe*y*BiB glasses was measured at 25 °C using a mobile contact angle meter PG-3 (Matsubo Corp., Tokyo, Japan) as a measure of hydrophobicity. The density was measured at 25 °C by Archimedes' method in order to conveniently estimate the distance between Fe ions linked by oxygen ions [11,12].

## 3. Results and Discussion

**Figure 1:**
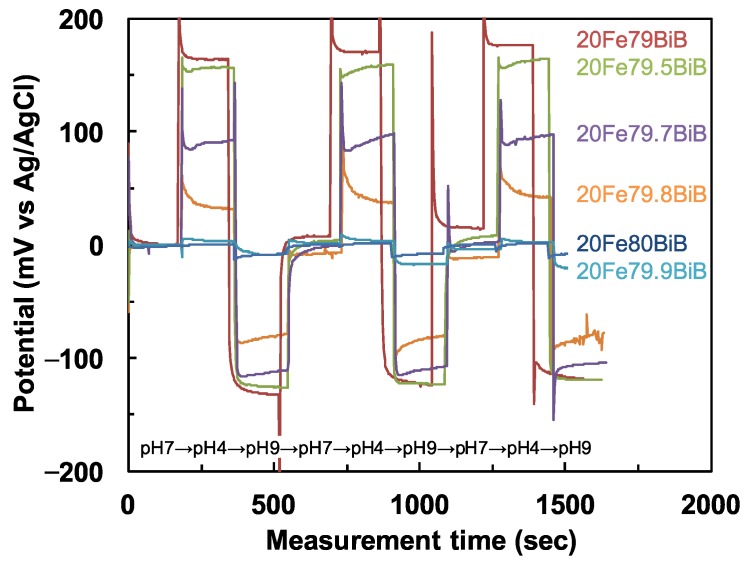
Change in potential with measurement time for 20Fe*y*BiB glasses in pH7, pH4 and pH9 buffer solutions.

**Figure 2:**
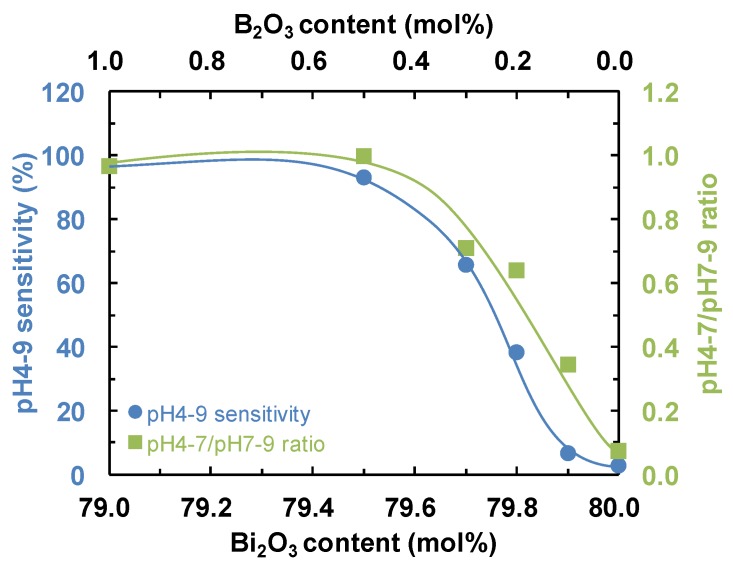
Dependence of pH4-9 sensitivity (left axis) between pH4 and pH9, and pH4-7/pH7-9 sensitivity ratio (right axis) on Bi_2_O_3_ and B_2_O_3_ contents for 20Fe*y*BiB glasses.

Figure 1 indicates the change in potential with measurement time for 20Fe*y*BiB glasses in pH7, pH4 and pH9 buffer solutions. When the Bi_2_O_3_ content increases to 79.7 mol %, the B_2_O_3_ content decreases to 0.3 mol %, the change in potential related to pH sensitivity drastically decreased. The dependence of the pH4-9 sensitivity (left axis) between pH4 and pH9, and the pH4-7/pH7-9 sensitivity ratio (right axis) on the Bi_2_O_3_ and B_2_O_3_ contents for the 20Fe*y*BiB glasses is shown in Figure 2. It can be seen from the left axis of Figure 2 that the pH4-9 sensitivity decreases to almost zero when the Bi_2_O_3_ content increases to 80 mol %. At this time, the pH4-7/pH7-9 ratio decreases with increasing Bi_2_O_3_ content. This corresponds to the decrease in H^+^ ion-selectivity for acid solutions (right axis of Figure 2).

**Figure 3:**
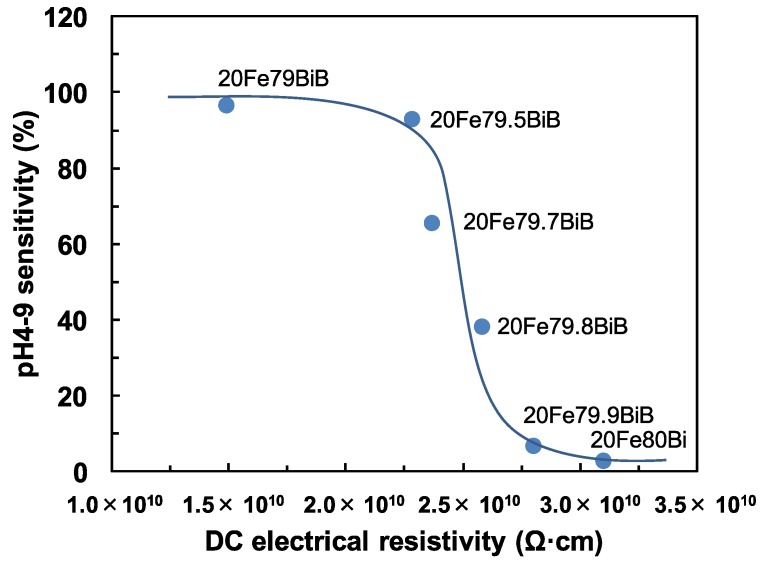
Relationship between pH4-9 sensitivity and DC electrical resistivity for 20Fe*y*BiB glasses.

Figure 3 presents the relationship between the pH4-9 sensitivity and DC electrical resistivity for the 20Fe*y*BiB glasses. The pH4-9 sensitivity decreased with increases in the DC electrical resistivity. In our previous work [10], the pH4-9 sensitivity of *x*Fe*y*BiB glasses was not so strongly affected by the glass compositions. However, a shortage of B_2_O_3_ seems to affect the pH4-9 sensitivity strongly in the present case. This suggests that a small amount of the B_2_O_3_ component (B-OH sites) may play an important role in the pH4-9 sensitivity. The reason for the decrease in pH4-9 sensitivity is complicated, because it decreases both with decreasing B_2_O_3_ content and with increasing DC electrical resistivity. Moreover, it should be noted that the decrease in pH4-7/pH7-9 ratio as a measure of H^+^ ion-selectivity for acid solutions is observed as the pH4-9 sensitivity decreases. Therefore, the pH4-9 sensitivity may be related to the specific glass compositions, such as the amount of B_2_O_3_, rather than the DC electrical resistivity.

**Table 1:** DC electrical resistivity, pH4-9 sensitivity, pH response time and contact angle for 20Fe*y*BiB glasses and related glasses.

Sample Name	DC Electrical Resistivity (Ω·cm)	pH4-9 Sensitivity (%)	pH Response Time (s)	Contact Angle (°)
70BiB	1.97 × 10^10^	- ^*1^	- ^*1^	93.3
10Fe50BiB	1.78 × 10^9^	(92.4) ^*2^	144	85.9
10Fe80BiB	1.68 × 10^9^	(92.3) ^*2^	>180	93.1
15Fe70BiB	6.96 × 10^8^	(97.3) ^*2^	39	93.6
20Fe20BiB	9.67 × 10^7^	88.7	15	87.5
20Fe50BiB	1.93 × 10^8^	92.6	10	87.9
20Fe60BiB	1.33 × 10^8^	92.6	9	93.5
20Fe70BiB	2.88 × 10^8^	90.2	10	94.0
20Fe79BiB	1.49 × 10^10^	96.6	22	82.8
20Fe79.5BiB	2.28 × 10^10^	92.9	18	78.8
20Fe79.7BiB	2.37 × 10^10^	65.5	20	80.1
20Fe79.8BiB	2.58 × 10^10^	38.3	15	79.8
20Fe79.9BiB	2.80 × 10^10^	6.7	(3) ^*3^	83.5
20Fe80Bi	3.10 × 10^10^	2.7	(6) ^*3^	79.0
70TP [6]	3.72 × 10^9^	80.7	12	58.8 (6.6) ^*4^
Reference (HORIBA)	1.06 × 10^10^	99.2	27	30.2

^*1^: Not determined; ^*2^: This value is determined using unstable potential owing to long pH response time; ^*3^: This value is short owing to low pH sensitivity; ^*4^: After UV irradiation.

Table 1 lists the DC electrical resistivity, pH4-9 sensitivity between pH4 and pH9, pH response time and contact angles of *x*Fe*y*BiB and related glasses [10] along with TP glass and a reference glass (HORIBA, Ltd., Kyoto, Japan). The DC electrical resistivity of 20Fe*y*BiB glasses changed from 9.67 × 10^7^ Ω·cm for 20Fe20BiB glass to 3.10 × 10^10^ Ω·cm for 20Fe80Bi glass (the second column in Table 1).

**Figure 4:**
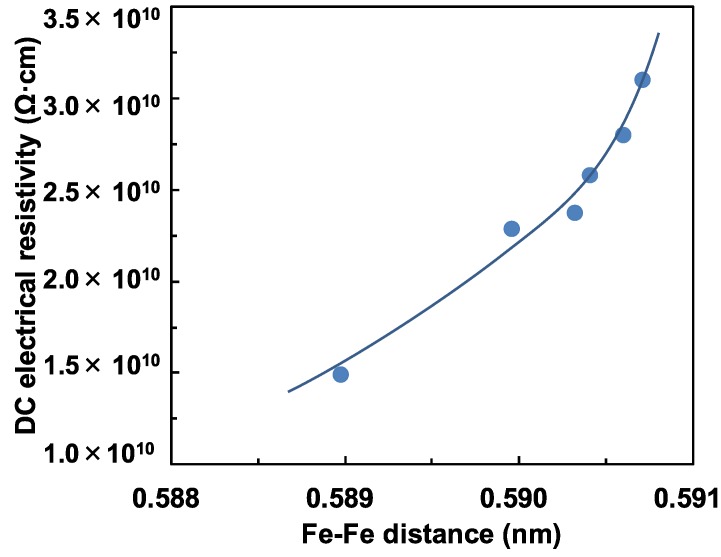
Dependence of DC electrical resistivity on Fe-Fe distance for 20Fe*y*BiB glasses.

Figure 4 shows the dependence of the DC electrical resistivity on the Fe-Fe distance for the 20Fe*y*BiB glasses. It is seen from Figure 4 that the DC electrical resistivity increases with increasing Fe-Fe distance (increasing Bi_2_O_3_ content). This is because electron hopping of from Fe^2+^ to Fe^3+^ via O^2^ ion becomes more difficult. This result is consistent with the data in [9,10].

We have previously reported that TP glasses with low DC electrical resistivity show a high pH4-9 sensitivity and short pH response time [6]. In the present work, the third column of Table 1 shows that *x*Fe*y*BiB glasses with a low DC electrical resistivity did not always show a high pH4-9 sensitivity. This suggests that the pH responsive sites (B-OH and or Bi-OH) may differ from the conductive sites (Fe-OH) in *x*Fe*y*BiB glasses, whereas the pH responsive and conductive sites are both Ti-OH in TP glasses. Taking the results of Figure 2 and Figure 3 into consideration, the most important pH responsive sites are B-OH in *x*Fe*y*BiB glasses.

On the other hand, the pH response time of 20Fe*y*BiB glasses (Table 1, column 4) tended to increase from 10 to 20 s on increasing the Bi_2_O_3_ content from 50 to 80 mol % (decreasing B_2_O_3_ content). We conclude that the pH response time of *x*Fe*y*BiB glasses is mainly determined by both (a) the dissociation rate of pH responsive sites, such as B-OH, at glass surfaces, and (b) the conduction rate of carriers (e) though the bulk glass. The latter is the predominant rate-determining process for the pH response of *x*Fe*y*BiB glasses, as well as in TP glasses [6]. However, the 20Fe20BiB glass with the lowest DC electrical resistivity did not show the shortest pH response time among *x*Fe*y*BiB glasses. This may suggest that the rate-determining process changes from a conduction process (bulk) to a pH response process (surface) with decreasing DC electrical resistivity in *x*Fe*y*BiB glasses.

Thus, a moderate amount of Fe_2_O_3_ and a small amount of B_2_O_3_ results in electronic conduction through the bulk glass and a pH response on glass surfaces, respectively. Because the remaining components can be selected freely, this result is a very useful in order to develop novel pH glass electrodes with functionalities such as self-cleaning [6], an anti-fouling ability [10], and so on.

So far, *x*Fe*y*BiB glasses with a Bi_2_O_3_ content larger than 60 mol % have shown contact angles higher than 90° [10]. Moreover, the fifth column in Table 1 reveals that a B_2_O_3_ concentration larger than 10 mol % is necessary for hydrophobicity. Thus, the present results suggest that using B_2_O_3_ as a glass former may play an important role in hydrophobicity as well as in pH4-9 sensitivity. Based on our results [7], Fe_2_O_3_-ZnO-B_2_O_3_ and Fe_2_O_3_-ZnO-Bi_2_O_3_-B_2_O_3_ glasses are candidate for pH glass electrodes with an anti-fouling property based on their hydrophobicity.

## 4. Conclusions

In the present study, the influence of Bi_2_O_3_ (or B_2_O_3_) content on the pH responsivity, electrical resistivity, and hydrophobicity was investigated for 20FeyBiB glasses in order to reveal the role of each glass component. The following results were obtained.

- The pH4-9 sensitivity drastically decreased with increasing the DC electrical resistivity or with decreasing the B_2_O_3_ content. In this case, a decrease in H^+^ ion-selectivity for acid solutions was also observed.
- The 20Fe*y*BiB glasses showed contact angles higher than 90° when they contain both more than 60 mol % Bi_2_O_3_ and more than 10 mol % B_2_O_3_. These results suggest that the use of B_2_O_3_ as a glass former plays an important role in pH4-9 sensitivity as well as in hydrophobicity.
- A moderate amount of Fe_2_O_3_ and a small amount of B_2_O_3_ respectively produces bulk electronic conduction and a pH response on glass surfaces. Because the remaining components of the glass can be selected freely, this discovery could prove very useful in developing novel pH glass electrodes that are self-cleaning and resist fouling.
